# Otorhinolaryngology Foundation: Three Decades of Excellence in Education, Research, and Scientific Advancement

**DOI:** 10.1055/s-0044-1801808

**Published:** 2025-02-10

**Authors:** Ricardo Ferreira Bento, Adilson Marcos Montefusco, Greice Vitória Garcia Ribeiro, Annita Luz Lacerda Lowndes, Adriana de Almeida Fozzati

**Affiliations:** 1 Department of Otorhinolaryngology, Universidade de São Paulo, São Paulo, SP, Brazil; 2 Fundação Otorrinolaringologia, São Paulo, SP, Brazil

**Keywords:** foundations, education, research, review, historical article, Brazil

## Abstract

**Introduction** The Otorhinolaryngology Foundation (FO), created in 1995, has played an important role in the advancement of otorhinolaryngology in Brazil, through initiatives in education, research, and service provision.

**Objective** To present the trajectory of FO's 30 years in public health and professional training, highlighting its contributions, historical milestones, and future challenges.

**Brief History** Created by the Center for Studies and Advanced Development in Otorhinolaryngology (CEDAO), the FO expanded its activities beyond academic support, becoming a national reference. Among its achievements, the commitment to the continuous improvement of teaching, research, publications, and provision of services to the community stands out. These initiatives include the promotion of courses and conferences that train thousands of professionals, the dissemination of knowledge through scientific publications, the development of innovative technologies, and the carrying out of campaigns and assistance actions aimed at the population.

**Final Considerations** The celebration of FO's 30th anniversary reaffirms its mission of excellence and innovation in the dissemination of knowledge in otorhinolaryngology in Brazil. The continuous commitment to improving its four fundamental pillars ensures quality in education, research, and service to society.

## Introduction

**Fig. 1:**
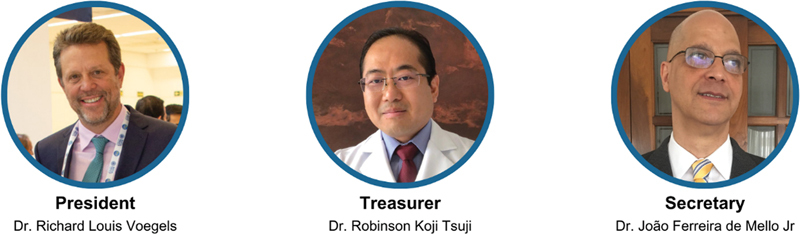
Photo of the current Board: From left to right: President: Dr. Richard Louis Voegels, Treasurer: Dr. Robinson Koji Tsuji, Secretary: Dr. João Ferreira de Mello Jr.

The year 2025 marks the 30th anniversary of the Otorhinolaryngology Foundation (FO), an institution that, since its creation in 1995, has played a fundamental role in the development of otorhinolaryngology in Brazil. Created by the Center for Studies and Advanced Development in Otorhinolaryngology (CEDAO), that supports the FO was founded to expand CEDAO's activities in the third sector, dedicating itself to health promotion and scientific advancement in otorhinolaryngology, speech-language pathology and audiology, psychology, and related areas. FO has established itself as a strategic link between health professionals, academics, and society. 1 The history of FO is marked by leaders committed to the advancement of public health and scientific development. Its boards have always been made up of professionals with outstanding academic, scientific, and clinical performance, who have contributed significantly to institutional strengthening. The leaders who created the FO was Prof. Dr. Ricado Bento, Prof. Dr. Aroldo Miniti and Dr. Sergio Garbi that registered approved the foundation in the Public Prosecutor's office of foundations from Sao Paulo State with a inicial donation from CEDAO of R$200,000 (Brazilian Reais). Among the presidents, Prof. Dr. Ivan Dieb Miziara, the first president of the FO and currently Full Professor of Legal Medicine at the Faculty of Medicine of the University of Sao Paulo (FMUSP); Ricardo Camargo (business enterprise); Prof. Dr. Luiz Ubirajara Sennes, who served from 2005 to 2014; and Prof. Dr. Richard Louis Voegels, current president ( Figure 1 ). 2 Furthermore, all boards had dedicated secretaries, treasurers, and administrators, who ensured administrative continuity throughout the different mandates ( Table 1 ).

**Table 1:** Previous boards of the Otolaryngology Foundation

**Eighth Board (2017 to 2019)** **President:** Dr. Richard Louis Voegels **Secretary:** Dr. Ronaldo Frizzarini **Tesoureiro:** Dr. Robinson Koji Tsuji	**Seventh Board (2014 to 2016)** **President:** Dr. Luiz Ubirajara Sennes **Secretário:** Dr. Ronaldo Frizzarini **Tesoureiro:** Dr. Richard Louis Voegels
**Sixth Board (2011 to 2013)** **President:** Dr. Luiz Ubirajara Sennes **Secretary:** Dr. Ronaldo Frizzarini **Tesoureiro:** Dr. Richard Louis Voegels	**Fifth Board (2008 to 2010)** **President:** Dr. Luiz Ubirajara Sennes **Secretary:** Dra. Francini Grecco de Melo Pádua **Tesoureiro:** Dr. Richard Louis Voegels
**Fourth Board (2005 to 2007)** **President:** Dr. Luiz Ubirajara Sennes **Secretary:** Dra. Francini Grecco de Melo Pádua **Treasurer:** Dr. Richard Louis Voegels	**Third Board (2002 to 2004)** **President:** Dr. Edigar Rezende de Almeida **Secretary:** Dr. Luiz Ubirajara Sennes **Treasurer:** Dr. Richard Louis Voegels
**Second Board (1999 to 2001)** **President:** Sr. Ricardo Ferraz de Camargo **Secretary:** Dr. Luiz Ubirajara Sennes **Treasurer:** Dr. Edigar Rezende de Almeida	**First Board (1995 to 1998)** **President:** Dr. Ivan Dieb Miziara **Secretary:** Dra. Priscila Bogar **Treasurer:** Dr. Luiz Ubirajara Sennes

**Fig. 2:**
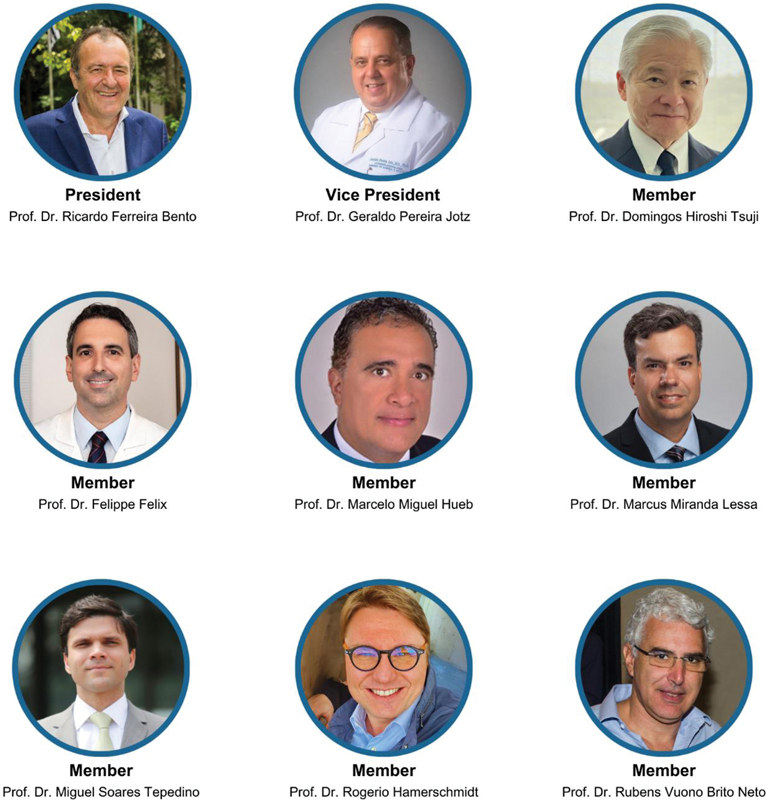
Board of Trustees of the Otorhinolaryngology Foundation (2024).

The FO Board of Trustees ( Figure 2 ) is composed of renowned specialists in the field, led by Prof. Dr. Ricardo Ferreira Bento as president and Prof. Dr. Geraldo Pereira Jotz as vice president. Currently, the Board includes Professors Dr. Domingos Hiroshi Tsuji, Dr. Felippe Felix, Dr. Marcelo Miguel Hueb, Dr. Marcus Miranda Lessa, Dr. Miguel Soares Tepedino, Dr. Rogério Hamerschmidt, and Dr. Rubens de Brito, all dedicated to advancing otolaryngology and fulfilling FO's mission. 2 It is important to highlight the founding members of the Board of Trustees, such as Professors Dr. Hélio Lessa (in memoriam), Dr. Marcos Mocellin, Dr. Shiro Tomita, and Dr. Roberto Meirelles, who made significant contributions over the years. Their dedication and work have left an invaluable legacy for the institution.

**Fig. 3:**
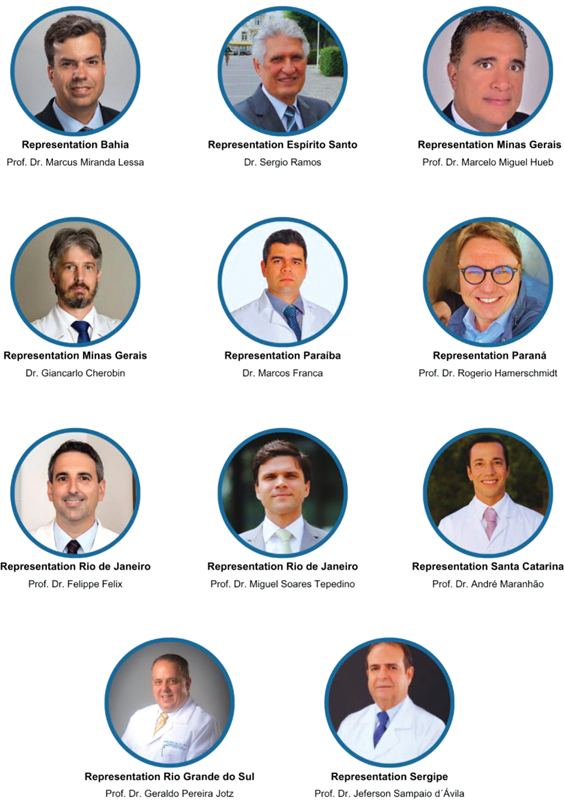
Representatives in Brazil (2024).

Recognizing the growing relevance of its actions and aiming to expand its institutional development, the Board of Trustees has broadened its activities by establishing representations in various regions of Brazil and forging strategic partnerships with prestigious institutions. Currently, FO has an extensive network of renowned representatives across the country ( Figure 3 ), strengthening its national presence and consolidating its operations in states such as Bahia, Espírito Santo, Minas Gerais, Paraíba, Paraná, Rio de Janeiro, Santa Catarina, Rio Grande do Sul, and Sergipe. Each representative plays an essential role in promoting FO's initiatives, contributing to the dissemination of knowledge and the advancement of the specialty in their respective regions. 2

The celebration of FO's 30th anniversary represents a significant milestone in the institution's transformative impact on public health and the population's quality of life. Over its history, FO has established itself as a reference in otorhinolaryngology, standing out for its scientific and technological contributions. Aligned with its mission to support and encourage the production of scientific knowledge with the applicability of technological innovation for community health, FO has successfully implemented programs focused on Comprehensive Care for the Hearing Impaired, alongside initiatives in Medical Research, Education, and Clinical and Surgical practices in otorhinolaryngology and related disciplines.

This historic milestone provides an opportunity to reflect on the challenges faced and achievements attained while renewing the institution's commitment to its four pillars: Teaching, Research, Publications, and Community Service.

## Teaching

### Training and Qualification Courses

FO stands out as a reference institution in the organization of multidisciplinary advanced training courses, solidifying its position as a center of excellence in the professional development of healthcare practitioners. Over its history, these courses have driven the technical and scientific advancement of thousands of participants, covering various areas of otorhinolaryngology, speech-language pathology and audiology, including the training of support teams.

In the field of otology, courses on temporal bone anatomy are particularly renowned, now incorporating the use of the Otobone® biomodel in 3D impression developed by the FO. This technology provides participants with an immersive practical experience, enabling a detailed study of each anatomical structure.

In rhinology, courses deeply explore nasal cavities, addressing everything from clinical aspects to advanced surgical techniques. Participants receive practical training in diagnostics, treatments, and innovative procedures, always under the supervision of highly qualified professionals.

In the area of bucopharyngolaryngology, the training stands out for including surgical procedures that are little explored in medical residencies and offices. Focusing on specialized training, these courses offer detailed recognition of anatomy and practical simulation of surgeries, expanding participants' skills.

In addition to these specific areas, FO organizes comprehensive courses ranging from introductory content to advanced approaches. These activities include diagnosis, interpretation of exams, and updated clinical practices, with opportunities to discuss clinical cases and interact with experienced professionals. These meetings promote the strengthening of an integrated and well-prepared medical community.

The great difference of FO is the integration between research and teaching, transforming patents into applied solutions that directly benefit clinical practice. These initiatives demonstrate FO's commitment to continuing education, excellence in teaching, and the advancement of clinical practices. Thus, FO consolidates its leadership position in the training of professionals and the development of otorhinolaryngology.

### Congress of the Otorhinolaryngology Foundation

**Fig. 4:**
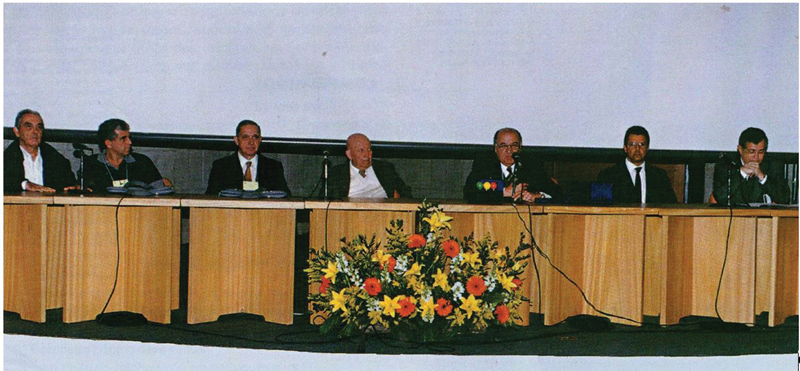
Opening session of the 1st FMUSP Otorhinolaryngology Congress, 1999.

In 1999, Prof. Ricardo Bento envisioned the First Otolaryngology Congress of the University of São Paulo (USP) to biennially bring together former students, residents, interns, and all those who had been part of the Otolaryngology Department at the Clinical Hospital, FMUSP. The inaugural event took place in São Paulo from June 18 to 20, 1999, gathering 1,700 participants ( Figure 4 ). Over time, the congress expanded beyond FMUSP, evolving into a national event and adopting the name Otolaryngology Congress of the Otolaryngology Foundation. 1

Under the leadership of professors Dr. Ricardo Ferreira Bento, and Dr. Richard Louis Voegels, and a highly qualified multidisciplinary team, the congress maintains a standard of excellence, integrating innovations and fostering essential discussions for the advancement of Otorhinolaryngology in Brazil. The impact of the event on the Brazilian Unified Health System (SUS) is notable, contributing to the constant updating of health professionals, the introduction of new technologies and clinical practices, in addition to strengthening of specialized care networks. The debates and advances promoted at the Congress result in direct benefits for the population served by the Brazilian public health system, especially in the prevention and rehabilitation of hearing and respiratory disabilities. 3

**Fig. 5:**
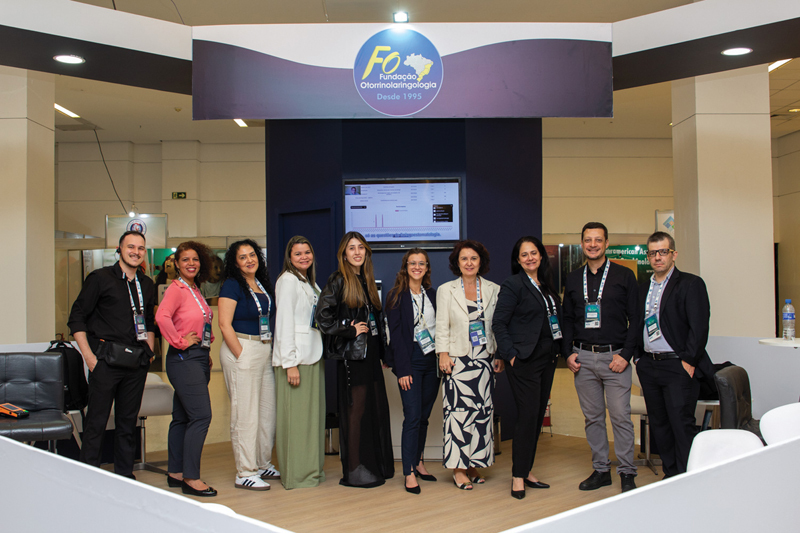
FO Team, 2024: João Assiz, Cleonice Souza, Nil Souza, Marília Rebouças, Greice Garcia, Annita Lowndes, Adriana Fozzati, Denise Cardenaz, Adilson Montefusco, and Flávio Doin (from left to right).

With 23 editions held, the FO Congress is a multidisciplinary event of great relevance, which brings together professionals from Otorhinolaryngology, Speech-Language Pathology and Audiology, and related areas. It stands out for promoting integrated teaching, improving diagnosis and patient care, and being a milestone in the exchange of scientific knowledge. 3 The organizational structure of the Congress is formed by the FO team, led by Adriana de Almeida Fozzati, administrative director, with support from: Adilson Montefusco, librarian, and editorial coordinator; Annita Lowndes, events coordinator; Greice Garcia, João Assiz, and Marília Rebouças, event analysts; Denise Cardenaz and Nil Souza, responsible for the financial department; Flávio Doin, graphic designer; Cleonice Souza, administrative assistant ( Figure 5 ).

In addition to the Congress, another prominent event organized by FO is the Hearing and Balance International Congress on Deafness, Cochlear Implants, Implantable Hearing Aids, and Balance Disorders first held in 2011. This biennial congress gathers national and international experts from various fields, including medicine, speech-language pathology and audiology, biology, engineering, psychology, and social work, with a focus on addressing deafness, and balance disorders and improving patients' quality of life. The multidisciplinary program features lectures, roundtable discussions, and practical workshops held at the Clinical Hospital, FMUSP. Among the hands-on activities, surgical training, rehabilitation, and advanced diagnostics are key highlights. 1

## Research

### CEDAO Institute

Founded in 1985 by professors Dr. Ricardo Ferreira Bento and Dr. Aroldo Miniti, its mission is to promote the development of health through teaching and research. Since its creation, CEDAO has established partnerships with public and private institutions, offering support in teaching, clinical, and surgical research, in addition to contributing to the development of new technologies and procedures. 1 4

Over the years, it has established itself as a reference in innovation in the area of Otorhinolaryngology, standing out for promoting product development, encouraging scientific publication, and promoting courses and conferences aimed at professional development in the health area. In 1995, the FO was established to expand CEDAO's activities, reaffirming its commitment to modernizing and expanding service in the specialty. 1 5

### ENT Library

**Fig. 6:**
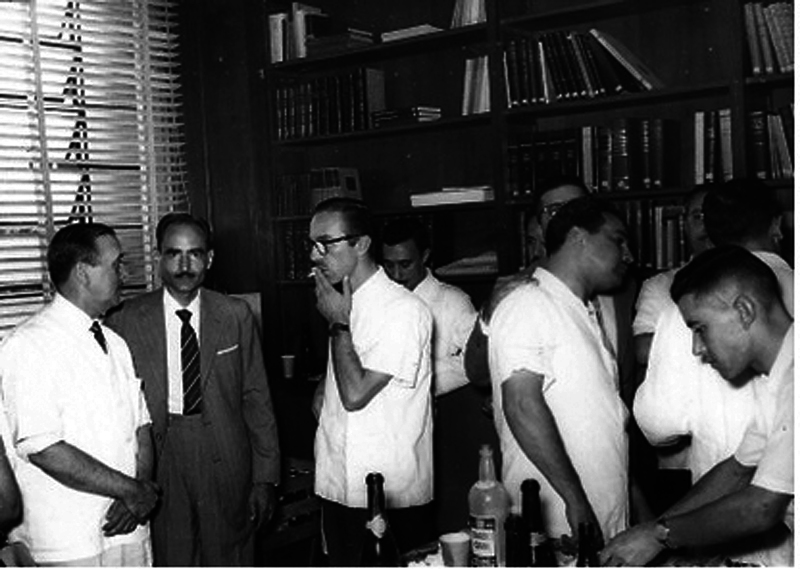
Library of the Discipline of Otorhinolaryngology in 1962, at the Central Institute of Clinical Hospital FMUSP.

**Fig. 7:**
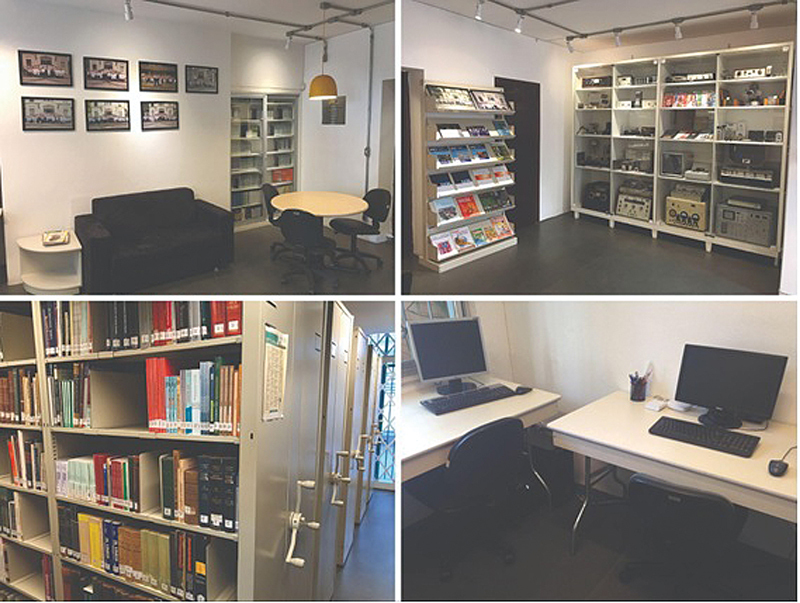
Current library at the Otorhinolaryngology Foundation.

Opened in 1948, the Otorhinolaryngology Library was installed at the Central Institute of Clinical Hospital FMUSP ( Figure 6 ). Its initial collection originated in the Santa Casa Otorhinolaryngology Service and, currently, the library is maintained by the FO ( Figure 7 ). The library holds over 1,500 works dedicated to otorhinolaryngology, including rare and unique volumes, some dating back to the 18th century. The collection is continuously expanded through the acquisition of new titles. Additionally, the library boasts an extensive collection of scientific journals, with subscriptions dating back to 1909. 1 In 2020, in response to the COVID-19 pandemic, the Digital Library of ENT was launched to provide remote support to researchers. The digital collection includes a wide range of academic resources, such as eBooks, conference proceedings, scientific articles, master's theses, and doctoral dissertations, solidifying its role as a reference platform for knowledge dissemination in the field. Access: https://digital.bibliotecaorl.org.br/

### Medical Investigation Laboratory (LIM-32)

LIM-32 began in 1994, thanks to the initiative of Professors Dr. Aroldo Miniti and Dr. Ricardo Bento. Its objective is to conduct experimental research with an emphasis on molecular and cellular biology, identification of new genes, functional studies, and cultivation of stem cells related to deafness. Under the responsibility of Prof. Dr. Jeanne Oiticica, her activities also include the use of induced pluripotent stem cells (iPS) and animal models to elucidate the pathophysiology of hearing loss and analysis of the cochlear proteome. Drs Karina Lezirovitz (geneticist) and Ana Carla Batissoco (pharmacist) actively lead these research initiatives in practice. These initiatives not only boost the production of scientific knowledge, but also encourage the development of doctoral theses, and significantly contribute to the training of new researchers in the field. 1 5 6

### Binaural Auditory Skills Laboratory (LHAB)

**Fig. 8:**
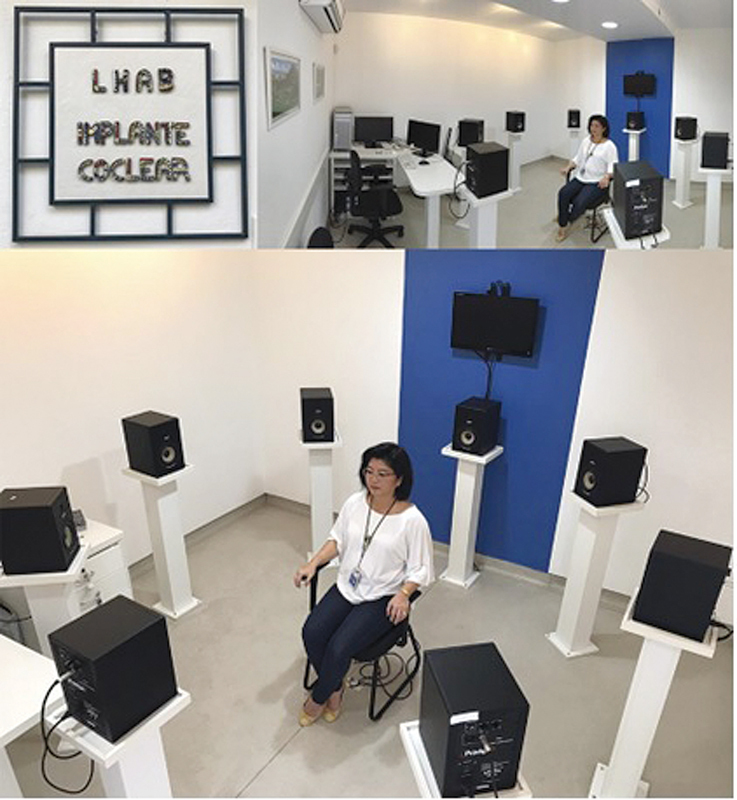
Binaural Auditory Skills Laboratory (LHAB).

LHAB, inaugurated in 2014, features a state-of-the-art setup consisting of eight interconnected speakers controlled by specialized software that manages the stimuli presented by each speaker ( Figure 8 ). This system allows for the assessment of auditory skills such as sound localization, figure-ground discrimination, the squelch effect, and others, following specifically developed protocols. The equipment is calibrated annually to ensure the precision of the auditory stimuli. This facility is unique in Brazil and supports a wide range of research studies involving patients using cochlear implants and hearing aids.

### Cochlear Implant Group

Comprising a multidisciplinary team, the Cochlear Implant Group provides high-quality auditory rehabilitation while maintaining a strong commitment to scientific research. The group explores innovative sound processing strategies and minimally invasive surgical techniques. As one of the largest cochlear implant centers in the world, it is the only facility in Brazil performing auditory brainstem implant surgeries, reaffirming its leadership in technological and medical advancements in auditory rehabilitation. 1

### Personal Sound Amplification Device (AASI)

In 2006, the patent registration process was initiated for the invention titled Generic Electronic Configuration for Digital Hearing Aids. On April 9, 2010, a Patent Licensing Agreement was formalized with Politec Importação e Comércio Ltda., granting the company the right to exploit the patent for production and commercialization for a period of 10 years. This agreement concluded in 2020. 1 2 Patent number: 060.5013-1.

### Audiotest

Developed in 2009, Audiotest was a hearing screening software available on iPhones until 2014. Created to carry out hearing tests, the application also provided entertainment to users. 1 2

### Telemedicine Hearing Assessment (Teleaudiometry)

Software that makes it possible to perform audiometry remotely, allowing self-audiometry in schools and automatically feeding data into the central system. 2 7

### Electronic and Digital Device for Active Noise Cancellation to Replace Audiometric Booths in Audiometry Exams and Related Tests

The electronic and digital device for active noise cancellation offers a portable alternative to traditional audiometric booths. Composed of an insulating acoustic cover, internal and external microphones, speakers emitting complementary signals, and a digital processing module, the device eliminates environmental noise, ensuring accuracy in audiological exams, even outside controlled environments. On July 20, 2010, a patent application was filed under the number 100.2508-1

### Short Hairpin RNA

Recombinant retrovirus comprises a vector for the transcription of a shRNA within a cell, in which the shRNA substantially inhibits the expression of a target gene. On 08/12/2014, the patent application was registered: BR 10 2014 019929 2.

### Otobone

Developed in 2016, Otobone is a biomodel of the Temporal Bone that emerged to assist in the training of otological surgeries. 2 8 Patent: BR 10.2015.032213-5.

### M-Scope

Developed in 2017, the M-Scope® kit (endoscope coupling adapter + portable LED light source) was designed for use with endoscopes and smartphones, aiming to assist in capturing, filming, and sharing images for general videoendoscopy. 2 Patent: BR 10.2019.076937-5.

### Study to Improve Public Hearing Health Policies in Brazil

This project aims to define care protocols for patients who use the SUS to receive hearing aids, in addition to care from an otologist and speech-language pathologist and audiologist. We received equipment from FAPESP and worked to make Reouvir the gold standard within the SUS. KPIs - Key Performance Indicators will be used jointly for all accredited centers. FAPESP Process: 2023/10201-5. 9

## Scholarly Output

### International Archives of Otorhinolaryngology

**Fig. 9:**
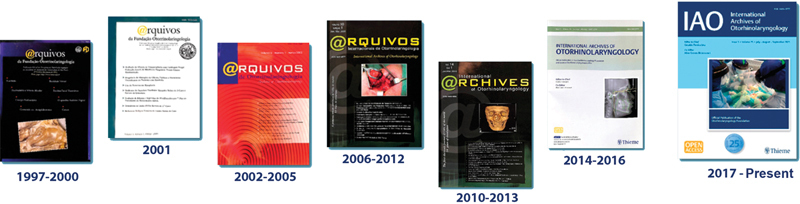
Covers of the International Archives of Otorhinolaryngology over the years (1997 to present).

The International Archives of Otorhinolaryngology (IAO) was founded in 1997, initially under the title "Arquivos da Fundação Otorhinolaryngologia" (ISSN: 1516-1528), by professors Dr. Ricardo Ferreira Bento and Dr. Aroldo Miniti. Since its first edition ( Figure 9 ), the journal has been the official publication of the Otorhinolaryngology Foundation. 10 The first editor-in-chief was Prof. Dr. Ricardo Ferreira Bento, followed by Prof. Dr. Tanit Ganz Sanchez (1999–2005), Prof. Dr. Marcelo Miguel Hueb (2006–2008), and Prof. Dr. Geraldo Pereira Jotz, current editor-in-chief who took over in 2009. Since 2013, Prof. Dr. Aline Bittencourt has served as co-editor. 11 In 2013, the journal began to be published by Thieme Medical Publishers, strengthening its presence in the international scenario of scientific publications in the medical field. 12 In 2023, the journal reached a significant milestone by obtaining its first JCR Impact Factor, highlighting its growing influence on the scientific scene. 13 In 2025, the IAO journal will celebrate 28 years of uninterrupted activities. In line with the principle that science must be accessible to everyone, since its first edition in 1997, it has adopted the open access model and exempts authors from any publication fees (No APC – Article Processing Charge). 10

### Books

**Fig. 10:**
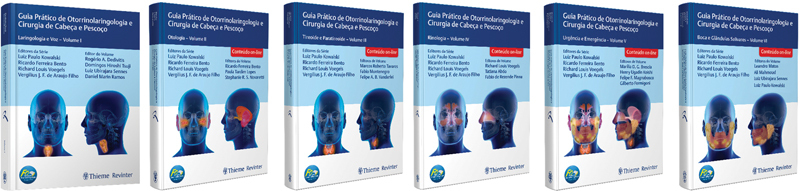
Collection: Practical Guide to Otolaryngology and Head and Neck Surgery. Vols. 1. Laryngology and Voice; 2. Otology; 3. Thyroid and Parathyroid; 4. Rhinology; 5. Emergencies and Urgencies; 6. Mouth and Salivary Glands (launching soon).

FO has published 27 books, both as an independent publisher and in partnership with other publishers, in addition to a collection consisting of five volumes. 1 14 15 16 17 18 19 20 21 22 23 24 25 26 27 28 29 30 31 32 33 34 35 36 37 38 39 These publications cover areas such as otology, rhinology, oropharyngeal surgery, and general topics. The *Guia Prático de Otorrinolaringologia e Cirurgia de Cabeça e Pescoço* (Practical Guide to Otorhinolaryngology and Head and Neck Surgery) collection ( Figure 10 ), the result of an unprecedented collaboration between the Otorhinolaryngology and Head and Neck Surgery departments at FMUSP, compiles, in five volumes published in partnership with Thieme Revinter, with two additional volumes in production, a comprehensive reference for these specialties. FO enhances the visibility of its publications through annual book launches at congresses, promoting the dissemination of scientific knowledge and contributing to academic and professional development.

### Magazines for General Public Information

**Fig. 11:**
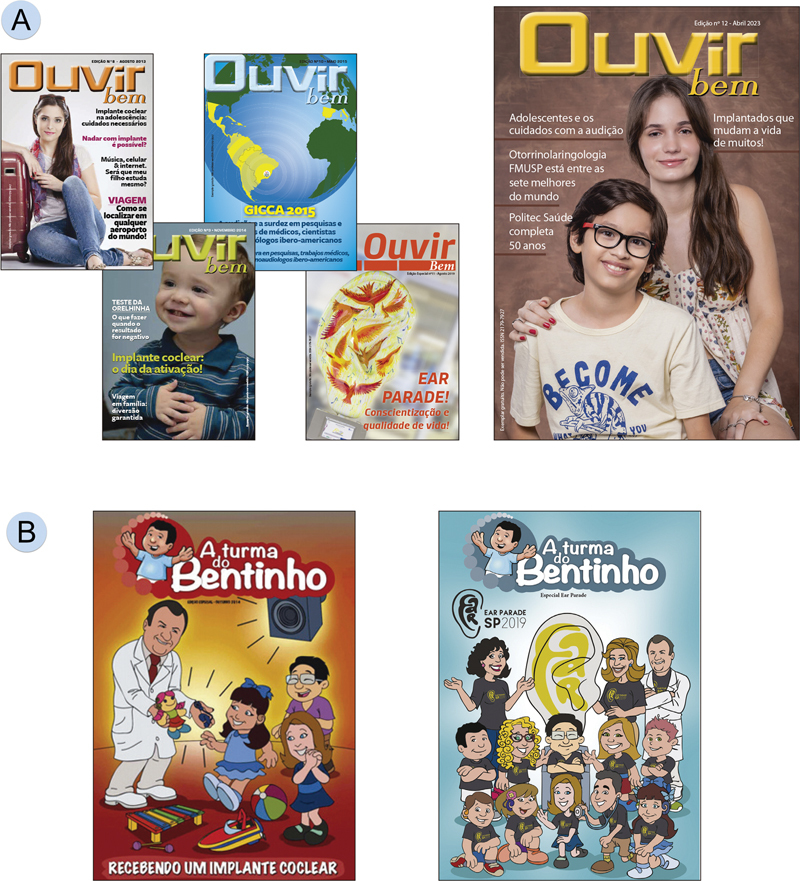
( **A** ) Ouvir Bem Magazine; ( **B** ) Turma do Bentinho.

Periodically, FO produces and distributes two publications free of charge, aimed at the public. One of them is a magazine targeted at adults ( Figure 11a ), while the other is a comic book designed for children ( Figure 11b ). Both aim to provide guidance on hearing care in an accessible and educational way. 2 5

## Public Service to the Community

Among the prominent projects of FO is the program "Quem ouve bem, aprende melhor" (Those Who Hear Well Learn Better), launched in 1999 in partnership with the Brazilian Society of Otology and the Federal Government. With a pioneering methodology, the program identified children with hearing loss in public schools, screening around 3 million students in 480 municipalities. In addition to raising awareness among parents, teachers, and students, it referred to confirmed cases of medical care and hearing aid adaptation. Today, it uses telemedicine technologies to expand its reach. 1 In 2002, the campaign was expanded under the title "Ouvir Bem para Aprender Melhor" (Hear Well to Learn Better), in partnership with Cia Belgo Mineira. Throughout its editions, the initiative was carried out in several cities in Minas Gerais.

The Reouvir Program, launched in 1999 and conceived by Dr. Sérgio Garbi and Dr. Mara Gândara, aims to demystify hearing loss in the elderly, raising awareness about the issue and contributing to auditory rehabilitation, focusing on improving the quality of life for older adults. Among its initiatives, the Employment Bank stands out, connecting people with hearing disabilities to the job market. The program also increased the acceptance of hearing aids for people of all ages, providing significant benefits with improved hearing. Additionally, it played an important role in combating prejudice, highlighting the transformative impact of auditory rehabilitation. 1 5

GAPZ (Support Group for People with Tinnitus), created in 1999, aims to guide and support people with tinnitus, promoting monthly meetings led by an experienced professional team. The group seeks to encourage the exchange of experiences between participants and offer up-to-date information on the topic. Currently, Dr. Jeanne Oiticica coordinates “Pílulas do GAPZ”, short lectures broadcast live on the FO YouTube channel. Each edition features the participation of an expert, who enriches the discussion about tinnitus and answers questions sent by the public. 1

The National Week to Prevent and Combat Deafness, held in November and December 2005, promoted activities aimed at raising awareness and caring for hearing health. During the event, there were lectures on topics such as ear infections and ear hygiene, the distribution of information materials in public places, and simple hearing screenings. Participants with suspected hearing loss underwent more detailed examinations at the FO and were referred for specific treatments. The initiative also stood out for its display of a giant ear, used to educate the public about how the ear works and the process of hearing. 2

From 2006 to 2009, FO provided free services to children at the Centro Educacional Sal da Terra, in Taboão da Serra, Brazil. The action, coordinated by Dr. Ronaldo Frizarini and Adriana Fozzati, included otorhinolaryngology consultations, otoemissions, audiometry, and nasofibroscopies. According to FO, early detection of hearing and respiratory problems is essential for children's learning. 2

In 2007, FO participated in the Health Fair with a stand at CPTM's Brás Station. During the event, doctors provided care and guidance to the population on otorhinolaryngological diseases, treating around 100 people. In addition, information leaflets were distributed, highlighting materials on allergic rhinitis. 2

In 2008, FO participated in the Global Action, held at CAT Paulo de Castro Correa, in Santos, Brazil. The event was attended by approximately 600 people, who received information about deafness and ENT diseases through explanatory folders. In addition, doctors used a giant ear model to demonstrate the necessary care for hearing health, promoting greater awareness among those present. 2

In 2012, free classes were held for the lay public on Combating and Preventing ENT Diseases, with monthly meetings dedicated to disseminating information on the prevention and treatment of diseases that affect the throat, larynx, and ear. The initiative was created to help the population care for their otorhinolaryngological health, promoting awareness and encouraging preventive practices. 2

The *Casa do Ouvir Bem* project was designed and put into practice by the Cochlear Implant Group in 2016, to help families of children using cochlear implants in the various aspects that influence the development of auditory and oral language skills, such as effective use of devices, routine, limits, and creation of a stimulating environment that could favor this development. Furthermore, the objective was also to create a channel of easy access and contact between the Cochlear Implant Group, the speech-language pathologists and audiologists, and the schools of these children. Over the years, dozens of families and children have been helped, benefiting from the guidance and the exchange of information between professionals, which has increased the chances of success with cochlear implants.

**Fig. 12:**
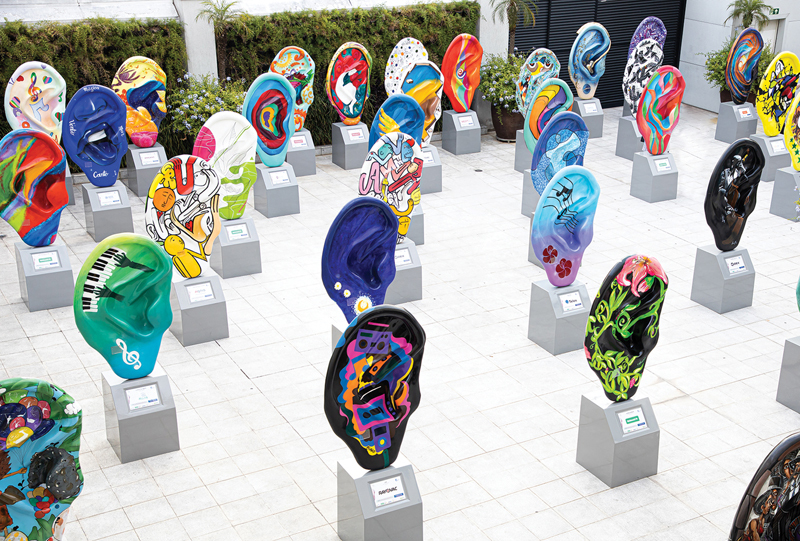
Custom Ear Sculptures.

Ear Parade, launched in 2019 as an initiative by FO in partnership with Prof. Dr. Ricardo Ferreira Bento, combined urban art and auditory health in an innovative way. The event featured more than 60 customized ear sculptures ( Figure 12 ), impacting millions of people and raising funds for auditory rehabilitation. Additionally, it promoted awareness about the importance of taking care of auditory health, with a focus on the prevention and treatment of hearing loss. 5 40

## Self-Sustaining Actions

### Audiology Center

The Audiology Center was created to rehabilitate the hearing of patients with different types of hearing loss, considering national and international scientific protocols and prioritizing the individualized adaptation of the patient. It offers state-of-the-art technology, regardless of the brand or device chosen. 5

## Future/Challenges

The future of FO is guided by a vision of innovation and accessibility, aiming to integrate education, research, and assistance to benefit diverse communities. It is expected that technological innovations will advance in a way that allows the creation of remote audiometry systems, and the development of apps equipped with cameras capable of capturing internal images of the nose, broadening the possibilities for remote diagnostics and making healthcare more accessible.

There is also the expectation that telemedicine will evolve significantly, enabling patients to perform basic clinical records and allowing diagnoses to be made remotely, especially for populations in situations of social vulnerability. The symbiosis between education, research, and technological innovation is seen as a central goal, with the potential to transform ideas into products and services focused on community assistance.

Another projected challenge is the adaptation of education to digital platforms, with greater use of artificial intelligence to enhance learning and expand access to hybrid and online courses. These initiatives aim not only at professional training but also at the democratization of health education.

It is also expected that the IAO journal will continue to increase its impact factor and be indexed in prestigious databases such as Medline, strengthening its academic and scientific impact. Additionally, the continuation of publishing and updating books in the fields of otolaryngology and related disciplines is projected.

Future projects include the creation of mobile clinics that serve underserved communities, providing preventive guidance and specialized diagnoses, with a focus on pediatric auditory health.

In research, the establishment of a Research and Development Center for Sensory Disabilities is projected, along with the expansion of national and international partnerships, and the strengthening of FO's Congress as a global reference. There is also the expectation of using data science, implementing strategies to increase social media presence, conducting market research, and strengthening academic partnerships that promote accessibility and excellence in training.

The development of technologies such as remote adaptation systems for hearing aids and the expansion of medical training with resources like the artificial bone developed by FO are seen as potential growth areas. Thus, FO's future is rooted in the expectation of advancements that promote health, innovation, and inclusion, consolidating its mission to contribute broadly and effectively to the community.

## Final Considerations

The celebration of the 30th anniversary of the Otorhinolaryngology Foundation marks a moment of reflection on its trajectory and planning for the future, reaffirming its commitment to the continuous improvement of its four fundamental pillars. In the field of teaching, FO has been promoting the training and development of professionals for more than 25 years through conferences and courses, such as the renowned Temporal Bone Dissection Courses, which have already had more than 150 editions. In the research area, it maintains partnerships with public and private institutions, offering support for teaching and the development of innovative projects. Furthermore, it preserves the collection of its Library, which since 1948 has provided support to researchers.

FO also stands out in scientific dissemination through the IAO journal, published in open access and without submission fees since 1997, and in the publication of renowned books in Brazilian medical literature. In addition, it distributes free educational materials to the public, providing guidance on hearing care. As part of providing services to the community, it supported several social programs such as *Quem Ouve Bem* , *Aprende Melhor* , which identifies hearing loss in children, and *Reouvir* , aimed at hearing rehabilitation for the elderly. It also organizes GAPZ, which offers support to patients with tinnitus and promotes initiatives such as the Ear Parade, which combines urban art and hearing health, raising awareness among millions of people and raising funds.

Looking to the future, FO seeks to continue innovating and strengthening its four pillars, to reach new levels of excellence. The celebration of 30 years represents a milestone in the renewal of commitment to education, science, and service to society.
